# Decreased plasma kallikrein activity is associated with reduced kidney function in individuals with type 1 diabetes

**DOI:** 10.1007/s00125-020-05144-1

**Published:** 2020-04-08

**Authors:** Mari-Anne Härma, Emma H. Dahlström, Niina Sandholm, Carol Forsblom, Per-Henrik Groop, Markku Lehto, on behalf of the FinnDiane Study Group

**Affiliations:** 1 Folkhälsan Institute of Genetics, Folkhälsan Research Center, Biomedicum Helsinki, Haartmaninkatu 8, 00290 Helsinki, Finland; 2 Abdominal Center Nephrology, University of Helsinki and Helsinki University Hospital, Helsinki, Finland; 3 Research Program for Clinical and Molecular Metabolism, Faculty of Medicine, University of Helsinki, Helsinki, Finland; 4 Department of Diabetes, Central Clinical School, Monash University, Melbourne, VIC Australia

**Keywords:** Diabetic nephropathy, Kidney function, Plasma kallikrein, Plasma kallikrein–kinin system, Type 1 diabetes

## Abstract

**Aims/hypothesis:**

Plasma kallikrein is the central mediator of the plasma kallikrein–kinin system, which is involved both in vascular control and thrombin formation cascades. The plasma kallikrein–kinin system has also been considered protective in pathological conditions, but the impact of plasma kallikreins on diabetic nephropathy remains unknown. The objective of this cross-sectional study was to explore the association of plasma kallikrein with diabetic nephropathy.

**Methods:**

We measured plasma kallikrein activity in 295 individuals with type 1 diabetes at various stages of diabetic nephropathy, and we tested the genetic association between the plasma kallikrein–kinin system and kidney function in 4400 individuals with type 1 diabetes.

**Results:**

Plasma kallikrein activity was associated with diabetes duration (*p* < 0.001) and eGFR (*p* < 0.001), and plasma kallikrein activity was lower with more advanced diabetic nephropathy, being lowest in individuals on dialysis. The minor alleles of the *KNG1* rs5030062 and rs710446 variants, which have previously been associated with increased plasma pre-kallikrein and/or factor XI (FXI) protein levels, were associated with higher eGFR (rs5030062 β = 0.03, *p* = 0.01; rs710446 β = 0.03, *p* = 0.005) in the FinnDiane cohort of 4400 individuals with type 1 diabetes.

**Conclusions/interpretation:**

Plasma kallikrein activity and genetic variants known to increase the plasma kallikrein level are associated with higher eGFR in individuals with type 1 diabetes, suggesting that plasma kallikrein might have a protective effect in diabetic nephropathy.

**Electronic supplementary material:**

The online version of this article (10.1007/s00125-020-05144-1) contains peer-reviewed but unedited supplementary material, which is available to authorised users.

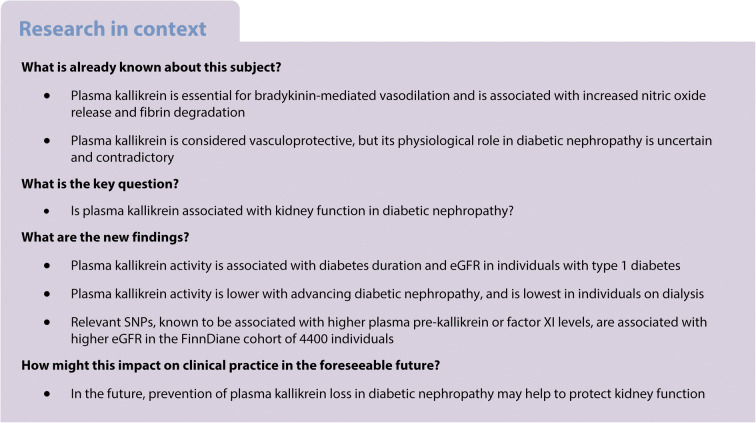



## Introduction

Finland has the highest incidence of type 1 diabetes in the world [1]. One-third of these individuals with type 1 diabetes are at high risk of the devastating complication diabetic nephropathy, which is associated with a manifold increased risk of cardiovascular disease and premature death. The pathogenesis of diabetic nephropathy is still not fully understood, but chronic inflammation, endothelial dysfunction and genetic propensity seem to play a role.

The kallikrein–kinin system (KKS) is considered part of the vasodilation regulatory system and is also involved in endothelial NO stimulation. Plasma kallikrein (encoded by the gene kallikrein B1[*KLKB1*]) has kininogenase activity through high-molecular-weight kininogen (HMWK) and it circulates in the blood as an inactive pre-kallikrein, bound to its cofactor HMWK. Cleavage of the kallikrein–HMWK complex leads to the release of the vasoactive peptide bradykinin, thus affecting vasodilation and inflammation, but, more importantly, it leads to increased NO release and bioavailability [2] and fibrin degradation [3], functions that may potentially be vasoprotective. Although it is widely accepted that the plasma KKS, as part of the contact-activated cascade, is locally assembled and profoundly silenced by various plasma inhibitors, accumulating evidence indicates that plasma KKS is activated by contact with an activated endothelial surface [4–7]. As kidneys have one of the most diverse endothelial cell populations amongst organs and some of the hallmarks of kidney disease are endothelial dysfunction and reduced NO synthesis and bioavailability, we aimed to evaluate plasma kallikrein activity in individuals with type 1 diabetes and diabetic nephropathy. We screened for previously published functional genetic variants that are known to affect the plasma KKS to assess their effect on kidney function in individuals with type 1 diabetes.

## Methods

### Participants

The nationwide Finnish Diabetic Nephropathy Study (FinnDiane) aims to identify genetic, environmental and clinical risk factors for diabetic complications such as diabetic nephropathy in adult individuals with type 1 diabetes. The study protocol was approved by the ethics committee of the Helsinki and Uusimaa Hospital District, and each participant gave written informed consent before the study. During the study visit, blood pressure was measured, blood samples (fasting or after a light breakfast) were collected and analysed for HbA_1c_ and serum creatinine by accredited hospital laboratory methods at the Helsinki University Central Hospital Laboratory (HUSLAB; www.hus.fi) or at the local study centres. eGFR was calculated using the Chronic Kidney Disease–Epidemiology Collaboration (CKD-EPI) equation and renal status was assessed using urinary albumin excretion rate and albumin/creatinine ratio. For details regarding diagnostic criteria and clinical assessments please refer to the Electronic supplementary material (ESM) Methods.

### Samples and storage

For this cross-sectional sub-study, plasma sample collection commenced in the year 2012, and all individuals visiting at the Helsinki University Hospital FinnDiane centre during 2012–2016 were included. Plasma citrate samples were collected from each participant into a 3 ml vacutainer containing 0.109 mol/l sodium citrate. To minimise protease auto-activation, samples were incubated for 30 min and then centrifuged at room temperature for 15 min at 2000 *g*. Plasma was collected and centrifuged again for 15 min at 2000 *g*. The final plasma samples were divided into 1 ml polystyrene tubes and stored at −80°C until analysis.

### Plasma kallikrein assay

The plasma kallikrein assay was done using a chromogenic substrate H-d-Pro-Phe-Arg-paranitroanilide (S-2302, Haemochrom Diagnostica, Essen, Germany). For a detailed description of the assay protocol and validation, please refer to the ESM Methods.

### Factor XI assay

We assessed factor XI (FXI) activity together with plasma kallikrein since the upstream coagulation factor factor XII (FXII) activates FXI in parallel with plasma kallikrein (ESM Fig. 1). The FXI assay was performed using a chromogenic substrate-based commercial assay (product no. COA0090; CoaChrom Factor XI, Coachrom Diagnostica, Maria Enzersdorf, Austria). For detailed information about the assay, please refer to the ESM methods.

### Genotyping and quality control

All available DNA samples (stored at −20°C) for the FinnDiane cohort underwent genome-wide genotyping using Illumina HumanCoreExome BeadChips (Illumina, San Diego, CA, USA) at the Genome Analysis and Technology Core (University of Virginia, Charlottesville, VA, USA) in 2016. After quality control, data on eGFR were available for 4400 of the participants eligible for this study, which included 294 individuals with measured plasma kallikrein activity. For details on the genotyping and quality control, please refer to the ESM Methods.

### SNP selection

We selected five previously identified SNPs (ESM Table 1) with established genome-wide significant associations with circulating FXII (*F12* rs1801020), plasma pre-kallikrein (*KLKB1* rs1511802, *KNG1* rs5030062) or FXI (*KNG1* rs5030062, *KNG1* rs710446, *F11* rs4253417, *F11* rs6842047, *F11* rs2289252) levels/activity. For details regarding the SNP selection criteria and characteristics, see ESM Methods and ESM Table 2.

### Statistical analyses

Plasma kallikrein values transformed using the natural logarithm for regression analyses. Multiple-group comparisons were performed using non-transformed data and the Kruskal–Wallis test, and two-group comparisons were performed using the Mann–Whitney *U* test. Spearman’s rank correlation and Pearson’s correlation were used for non-parametric and parametric data correlations, respectively. Logistic and linear regression analyses were used to test associations between individual SNPs and categorical and continuous variables, respectively. A *p* value <0.05 was considered statistically significant. For statistical methods for genetic analyses and power analyses, please refer to the ESM Methods.

## Results

### Participant characteristics

In the study cohort of 295 individuals with measured plasma kallikrein and FXI activity, 163 were men (55.3%). The mean (±SD) age was 47.2 ± 12.5 years and the mean duration of diabetes was 30.2 ± 11.9 years. The study cohort was divided into normoalbuminuria (*n* = 165), microalbuminuria (*n* = 41), macroalbuminuria (*n* = 37) and end-stage renal disease (ESRD, *n* = 52) (for category thresholds, see ESM Methods, Participants). For more details, see ESM Results and ESM Table 3.

### Impact of age, diabetes duration and medication on plasma kallikrein activity

In individuals with normoalbuminuria, both plasma kallikrein and FXI activity correlated negatively with age (*r* = −0.29, *p* < 0.001 and *r* = −0.21, *p* = 0.006, respectively), but only plasma kallikrein activity correlated negatively with diabetes duration (*r* = −0.27, *p* < 0.001).

Because the renin–angiotensin–aldosterone system (RAAS) and plasma KKS are interconnected systems, several antihypertensive medications could impact plasma kallikrein activity. In line with this, we observed an association between plasma kallikrein activity and RAAS-blocker therapy (β = −0.32, *p* = 0.03, see ESM Results for further details). RAAS-blocker therapy was thus accounted for in further regression analyses.

### Plasma kallikrein and FXI activity in individuals with diabetic nephropathy

**Fig. 1:**
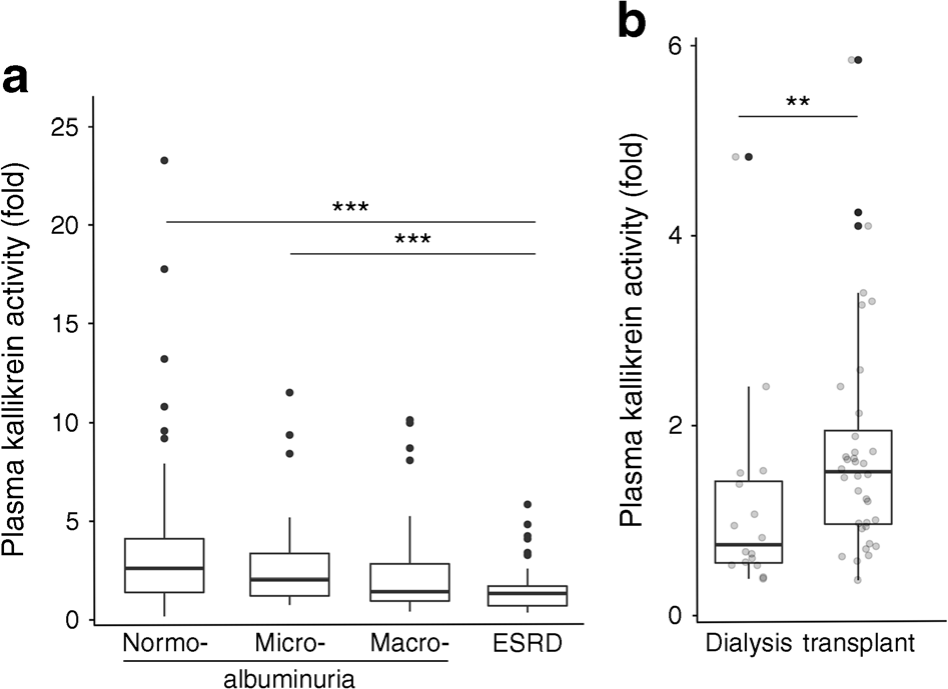
(**a**) Plasma kallikrein activity decreases by advancing stage of diabetic nephropathy in individuals with type 1 diabetes. Normoalbuminuria, *n* = 165; microalbuminuria, *n* = 41; macroalbuminuria, *n* = 37; ESRD, *n* = 52. (**b**) Plasma kallikrein activity in individuals with ESRD. Individuals on dialysis, *n* = 16; individuals with a kidney transplant, *n* = 36. Plasma kallikrein activity is expressed as fold change vs healthy control plasma samples. Box plots represent median, interquartile range and upper and lower quartile of plasma kallikrein activity levels. In (**b**), the grey circles represent individual data points, the black dots represent outliers. *p < 0.05, **p < 0.01, ***p < 0.001

Plasma kallikrein activity decreased with more advanced stages of diabetic nephropathy: normoalbuminuria (median 2.53, [IQR 1.37–4.23]; *n* = 165), microalbuminuria (2.04 [1.23–3.40]; *n* = 41), macroalbuminuria (1.31 [0.91–2.81]; *n* = 37), and ESRD (1.23 [0.66–1.72]; *n* = 52; *p* < 0.001) (Fig. 1a). Plasma kallikrein activity correlated positively with eGFR (*r* = 0.34; *p* < 0.001). This association remained after adjustment for age, sex, diabetes duration, waist circumference, HbA_1c_ and RAAS-blocker therapy (β = 0.24 [95% CI 0.14, 0.34], *p* < 0.001). Within the ESRD group, plasma kallikrein activity was lower in individuals on dialysis (median 0.74, [IQR 0.55–1.41], *n* = 16) than in kidney transplant recipients (1.51 [0.96–1.95]; *p* = 0.008, *n* = 36) (Fig. 1b). FXI activity did not differ within the ESRD group and was not associated with eGFR (data not shown).

### Plasma kallikrein and genetic variants

Genetic associations (adjusted for age, sex and diabetes duration) between selected SNPs and kallikrein activity were confirmed in the primary study cohort of 295 individuals. Altogether, three SNPs (*KNG1* rs5030062, rs710446 and *F12* rs1801020) were associated with plasma kallikrein activity (Table 1, ESM Results and ESM Fig. 2a, b) and selected for further analyses in the larger cohort of 4400 individuals (adjusted for age, sex, diabetes duration, RAAS-blocker therapy and the two first principal components). In these analyses, the minor allele of *KNG1* rs5030062 and rs710446 were additively associated with higher eGFR (rs5030062 β = 0.03, *p* = 0.01; rs710446 β = 0.03, *p* = 0.005). The association remained after removing individuals who had received kidney transplants ([*n* = 271] rs5030062 β = 0.02, *p* = 0.05; rs710446 β = 0.03, *p* = 0.01) and after removing first-degree relatives ([*n* = 182] rs5030062 β = 0.03, *p* = 0.02; rs710446 β = 0.03, *p* = 0.01). For expanded results on the associations between kallikrein and genetic variants and for results on the genetic associations with blood pressure and 24 h urine sodium, please refer to the ESM results.

**Table 1:** Plasma kallikrein activity associations

Chr.	SNP	Gene	Trait	EA	EAF	Plasma kallikrein	
						β (95% CI)	*p* value
3	rs5030062	*KNG1*	Factor XI/Pre-kallikrein	C	0.35	0.25 (0.11, 0.38)	3.9 × 10^−4^
3	rs710446	*KNG1*	Factor XI	C	0.37	0.24 (0.11, 0.37)	3.5 × 10^−4^
4	rs1511802	*KLKB1*	Pre-kallikrein	C	0.44	0.12 (−0.02, 0.25)	0.085
4	rs6842047	*KLKB1*	Factor XI	A	0.09	−0.02 (−0.23, 0.18)	0.81
4	rs4253417	*F11*	Factor XI	C	0.45	0.10 (−0.03, 0.23)	0.13
4	rs2289252	*F11*	Factor XI	T	0.43	0.09 (−0.04, 0.21)	0.19
5	rs1801020	*F12*	Factor XII	A	0.25	−0.63 (−0.77, −0.50)	3.2 × 10^−17^

Chr., chromosome; EA, effect allele (= minor allele); EAF, effect allele frequency; associations are adjusted for age, sex and diabetes duration. A Bonferroni-corrected *p* value of <0.007 was considered statistically significant

## Discussion

In this study we investigated the association of plasma kallikrein with kidney function and diabetic nephropathy in 295 individuals with type 1 diabetes. We showed that plasma kallikrein activity was associated with diabetes duration and eGFR and that the activity was lower in more advanced diabetic nephropathy, being lowest in individuals on dialysis. Furthermore, the minor alleles of the *KNG1* rs5030062 and rs710446 variants, which are associated with increased plasma pre-kallikrein and/or FXI levels, were associated with higher eGFR in the FinnDiane cohort of 4400 individuals with type 1 diabetes.

Irregularities in plasma KKS homeostasis have been demonstrated in individuals with type 1 diabetes and diabetic nephropathy [8–10], but the reason behind the decline in plasma kallikrein activity in our study is unknown. As plasma kallikrein circulates in complex with HMW-kininogen (280 kDa), it is unlikely to be filtered out when bound to its cofactor. However, when the plasma kallikrein–HMW-kininogen complex is cleaved, e.g. in response to damaged endothelium, then active plasma kallikrein (89 kDa) could be excreted in the urine [11], as the damage to the glomerular basement membrane increases. This could potentially result in reduced concentrations of the intact plasma kallikrein–HMW-kininogen complex in circulation.

We observed that plasma kallikrein activity was lower in individuals on dialysis compared with those who had received a kidney transplant. As individuals on dialysis have the most damage to their endothelium, lower plasma kallikrein activity would be expected in these individuals than in the kidney transplant recipients.

However, whether reduced plasma kallikrein activity is beneficial or deleterious in diabetic nephropathy is unknown. It is generally understood that the KKS has a protective effect under physiological and pathophysiological conditions [12–14], and plasma kallikrein has been associated with increased NO release and fibrin degradation, which are functions relevant to vascular protection. Consistent with these findings, *KNG1* rs5030062 and rs710446, which are known to increase plasma pre-kallikrein and/or FXI levels, associated with both plasma kallikrein activity and with increased eGFR in this study.

Our study was subject to certain limitations. The number of participants with available plasma samples was relatively small and therefore the study was not powered to detect any subtle changes that might exist. However, the observed effect size of the association between plasma kallikrein activity and eGFR was high enough to be able to detect a difference with 99% power. In addition, plasma kallikrein and FXI protein levels were not quantified in this study.

In this study we show that lower plasma kallikrein activity levels accompany more severe diabetic nephropathy and lower eGFR and that a genetic variant known to result in a higher plasma pre-kallikrein level is associated with higher eGFR. We propose that plasma kallikrein might have a protective effect on kidney function in type 1 diabetes, but prospective studies are needed to evaluate whether individuals with lower plasma kallikrein activity have worse kidney disease outcomes.

## Electronic supplementary material

ESM 1 (PDF 509 kb)

## Data Availability

The datasets generated and analysed in this study are not publicly available due to the local legislation and the written consents of the study participants, which do not allow sharing individual-level phenotype data.

## Abbreviations

ESRD: End-stage renal disease
FXI: Factor XI
FXII: Factor XII
FinnDiane: Finnish Diabetic Nephropathy Study
HMWK: High-molecular-weight kininogen
KKS: Kallikrein–kinin system
RAAS: Renin–angiotensin–aldosterone system

## Authors’ relationships and activities

P-H.G. has received research grants from Eli Lilly and Roche, lecture fees from Astellas Pharma, Astra Zeneca, Boehringer-Ingelheim, Eli Lilly, Elo Water, Genzyme, MSD, Mundipharma, Novartis, Novo Nordisk and Sanofi. He is an advisory board member for AbbVie, Astellas Pharma, AstraZeneca, Boehringer-Ingelheim, Eli Lilly, Janssen, Medscape, MSD, Mundipharma, Novartis, Novo Nordisk and Sanofi. All other authors declare that there are no relationships or activities that might bias, or be perceived to bias, their work.
