# Supplementary material for: Decreased plasma kallikrein activity is associated with reduced kidney function in individuals with type 1 diabetes
Source: Diabetologia. 2020 Apr 8;63(7):1349–54. doi: 10.1007/s00125-020-05144-1 (PMC7286847; doi:10.1007/s00125-020-05144-1)
Supplement: Supplementary file 1 — (PDF 509 kb) [file 125_2020_5144_MOESM1_ESM.pdf]

Electronic supplementary material (ESM)

Decreased plasma kallikrein activity is associated with reduced kidney function in individuals with type 1 diabetes

Methods

Participants. All study participants were Finnish adult individuals with type 1 diabetes. The diagnosis of type 1 diabetes for each participant was made by his/her attending physician at the time of diabetes onset according to national evidence-based clinical practice guidelines. We further refined the diagnosis of type 1 diabetes by requiring an onset of the disease before the age of 40 years, and permanent insulin treatment initiated within one year of diagnosis. During the FinnDiane study visit, participants underwent a clinical examination and completed standardized questionnaires regarding health and medical history in collaboration with the attending physician/nurse. Anthropometric measurements (waist circumference, weight, height and hip circumference) and blood pressure measurements were performed by a trained nurse. Blood pressure was measured two times with a two-minute interval in the sitting position after a ten-minute rest and the mean values of these two measurements were used in the analyses. We calculated the mean arterial pressure as 1/3 systolic blood pressure + 2/3 diastolic blood pressure in mmHg.

Renal status was assessed by the albumin excretion rate (AER) in two out of three consecutive timed overnight or 24h urine sample collections or by the albumin-to-creatinine ratio (ACR) in morning spot urine samples. The following criteria were used: normoalbuminuria AER<20 μg/min or <30 mg/24h or ACR <2.5 mg/mmol (for men) and <3.5 mg/mmol (for women); microalbuminuria AER≥20 and <200 μg/min or ≥30 and <300 mg/24h or ACR≥2.5 and <25 mg/mmol (for men) and ≥3.5 and <35 mg/mmol (for women); macroalbuminuria AER≥200

µg/min or≥300 mg/24h or ACR≥25 (for men) and ≥35 mg/mmol (for women), and end-stage renal disease (ESRD). The ESRD group comprised of patients having received a kidney transplant or undergoing dialysis. DN was defined as microalbuminuria, macroalbuminuria or ESRD. The eGFR was calculated using the CKD-EPI equation[1]. The main study cohort of 295 individuals was divided into four groups based on AER or ACR: normoalbuminuria (n=165), microalbuminuria (n=41), macroalbuminuria (n=37) and ESRD (n=52, from which 36 individuals had received kidney transplantation, and 16 individuals were on dialysis).

Plasma kallikrein assay. Plasma citrate samples were thawed promptly to +37˚C and diluted 1:5 in a warm buffer (20 mM Hepes, 140 mM NaCl) to minimize spontaneous activation. Diluted plasma samples (50 µl) were pipetted to 96-well Nunc plates (Thermo Fisher Scientific, USA) in duplicates. Samples were activated with a 50 µl of activator solution [35% acetone, 1µg/mL dextran sulfate MW 500 000 (17-0340-02, Pharmacia, Sweden)]. Plates were incubated at 37˚C for 15 min. Following activation, 50 µl of chromogenic substrate H-D-Pro-Phe-Arg-paranitroanilide (S-2302, Haemochrom Diagnostica, Essen, Germany) was added in the concentration of 0.1 mM, and the absorbance values were recorded at 405 nm at 37˚C, with a 1-min interval for 60 min. Results are expressed as baseline value subtracted from the end-point value, converted to relative units against healthy control plasma samples (n=3). The healthy control samples were generous donations by the FinnDiane researchers. The intra-assay CV was <10% and inter-assay CV<10%.

Measured sample baseline values were similar (0.13±0.03), and there was no statistical difference between DN groups (p=0.3). Assay specificity was validated with purified plasma kallikrein (420307-50UG, Merck, Germany) and FXII (233490-500UG, Merck, Germany) proteins and plasma kallikrein specific inhibition tests with soybean trypsin inhibitor (T6522-25MG, Merck, Germany) (data not shown). After the incubation of plasma samples with dextran sulfate, adding soybean trypsin inhibitor cleared all detectable plasma-kallikrein

activity signal. Plasma-kallikrein activation via dextran sulfate is dependent on available FXII, and FXII and plasma-kallikrein activities correlated in a limited pilot test (r=0.88; p=0.004; n=8) where FXII activity was measured (COA0068 CoaChrom Factor XII, Coachrom Diagnostica, Maria Enzersdorf, Austria) in parallel with plasma kallikrein activity. Plasma-kallikrein in serum samples, after the additional activation of dextran sulfate, yielded 81% higher activity compared to activated plasma from the same individuals, and activated kallikrein in serum samples correlated with activated kallikrein in plasma samples (r=0.81; p=0.05; n=6). It can be concluded, that dextran sulfate activation in plasma does not reflect the total available plasma kallikrein activity; instead, it reflected the underlying plasma kallikrein potential after induction by a similar activator. As this study intended to evaluate the differences of the physiologically inhibited plasma kallikrein in the context of diabetic nephropathy, measuring the protease activity under inhibitory conditions should resemble physiologic conditions more than quantifying the total protein.

Factor XI (FXI) assay. In parallel with the plasma kallikrein assay, the same samples were prepared for the FXI assay according to the manufacturer's protocol, and FXI activity was measured following the microtiter method (COA0090 CoaChrom Factor XI, Coachrom Diagnostica, Maria Enzersdorf, Austria). Absorbance values were recorded at 405 nm at 37˚C, with 1-minute intervals for 60 min. The inter-assay CV was <10%. We expressed the results as baseline value subtracted from the end-point value and converted to units/mL against normal plasma standard.

Genotyping and quality control. Genotyping was performed in three batches and quality control as well as genotype imputation was performed as previously described [2]. Batch one (5,088 FinnDiane samples) was genotyped using the HumanCoreExome Bead array 12 v. 1.0. Batch two (716 FinnDiane samples) was genotyped using the HumanCoreExome Bead array

12 v. 1.1. Batch three (91 was FinnDiane samples and 360 non-type 1 diabetes samples) was genotyped using the HumanCoreExome Bead array 24 v.1.0.

After genotyping, SNPs were filtered based on SNP call rate, deviation from Hardy-Weinberg equilibrium, MAF (<1%), Mendelian inconsistency, and allele frequency difference with the 1000 Genomes European (EUR) population (>20% [if MAF≥5%] or >5% [if MAF<5%]). The remaining SNPs (n= 316,899 SNPs) were used for imputation with the Minimac3/Minimac3-omp v1.0.14 software (1000 Genomes EUR phase 3, version 5). After SNP quality control, sample quality control was completed (sample genotyping rate <0.95, extreme heterozygosity, sample mix-ups, and removal of genetic outliers) resulting in 6,019 individuals. Part of these individuals was initially recruited by the Finnish Institute of Health and Welfare (16.7%, n=863) and was not included in the current study, resulting in 5,161 participants with available genome-wide genotyping data. We further excluded those that did not meet the type 1 diabetes criteria in this study (diabetes onset age <40 years and insulin treatment within 1 year; n=283) and patients with missing data for age, diabetes duration and sex (n= 292) as well as RAAS blocker therapy (n=66) resulting in 4,520 individuals. Of these 4,520 individuals, we had data on eGFR for n=4400 (2.7% missing), diabetic nephropathy for n=4349 (3.8% missing), systolic blood pressure for n= 4414 (2.3% missing), diastolic blood pressure for n=4410 (2.4% missing), and for 24h urine sodium concentration for n=2491 (44.9% missing). The 10 first principal components were calculated with the EIGENSTRAT, version 3.0.

SNP selection. To select SNPs affecting the KKS system (ESM Fig. 1), we used the curated literature-based GWAS catalogue (https://www.ebi.ac.uk/gwas/) and searched for genome-wide significant genetic variants for FXII, plasma kallikrein and FXI using the search words ‘plasma kallikrein’, ‘Factor XI’ and ‘Factor XII’. We then selected SNPs associations performed in studies with n≥500 individuals. The search resulted in two SNPs for plasma kallikrein (*KLBK1* rs1511802, *KNG1* rs5030062), and five SNPs for factor XI (*KNG1*

rs710446, *F11* rs4253417, *F11* rs2289252 and *KNG1* rs5030062, ESM Table 1). There were no results for factor XII. However, *F12* rs1801020 has previously been established to alter factor XII protein levels, therefore this SNP was additionally included in the analyses. We extracted genotypes from the FinnDiane GWAS for the selected SNPs. We converted imputed genotypes to most likely genotypes using a 90% threshold for the genotype posterior probability. Deviations from the Hardy-Weinberg Equilibrium (HWE) were assessed using the exact test statistics in PLINK1.9.[3]. None of the SNPs deviated significantly from the HWE (ESM Table 2).

Statistical analyses. Five individuals (1.7%) with measured kallikrein and FXI activity had missing data for some variables and were thus excluded from the analysis. Genetic associations were tested using an additive model for the SNP and the associations were adjusted for age, sex, diabetes duration, renin-angiotensin-aldosterone system (RAAS) blocker therapy and the two first principal components calculated based on the genome-wide genotyping data to account for population substructure in Finland [2]. Genetic association and epistasis analyses were done in PLINK, and other statistical analyses were done in R[3]. For genetic comparisons, Bonferroni correction was applied.

We calculated the power of the genetic analyses using QUANTO [4] and the power of the other analyses using R (WebPower package). Power analyses were performed as *post hoc* analyses for the observed effect sizes in the study. In the genetic analyses, our study had 73% power to detect a significant association between *KNG1* rs5030063 and eGFR with the observed effect size of β=0.02 and 74 % power to detect a significant association between *KNG1* rs710446 and eGFR with the observed effect size of β=0.03. For the genetic analyses with 24h urine sodium concentration, we had 54 % power to detect an association between *F12* rs1801020 and 24h urine sodium concentration with the observed effect size of β=2.15. In the non-genetic association analyses performed in the 295 individuals with measured plasma kallikrein activity

and factor XI activity, we had 99% power to detect an association between plasma kallikrein activity and eGFR with the observed effect size of β=0.24 (Cohen effect estimate f2= 0.08) and 27% power to detect a significant association study between factor XI activity and eGFR with the observed effect size of β=0.36 (Cohen effect estimate f2=0.007). In the analyses with plasma kallikrein activity and blood pressure (β=0.001), we had 6% power to detect such subtle changes (Cohen estimate f2=0.0004).

Results

Patient Characteristics. The proportion of women was significantly lower among patients with macroalbuminuria and ESRD. Within each DN group, there were no differences in age and diabetes duration between genders. The subset of 295 individuals within the assigned renal status groups (except ESRD group) were on average five years older and had five years longer diabetes duration compared to the larger FinnDiane cohort of 4400 individuals (data not shown). Clinical characteristics for the sub-cohort are presented in ESM Table 3.

Impact of age, diabetes duration and medication on plasma kallikrein activity. Cross-talk between the KKS and the RAAS has been discussed [5–8], suggesting that KKS has a counterbalancing role to RAAS. Furthermore, plasma kallikrein and *F12* rs1801020 are involved in the RAAS in healthy individuals [9]. By measuring plasma kallikrein activity, one can get some information about the functionality of this biologically inhibited protease under pathological conditions, and it is possible, that the RAAS-blocker therapy adds to this biological inhibition, preventing plasma kallikrein from functioning correctly.

Individuals who were on angiotensin-2 receptor blocker (AT2RB) therapy had in general lower plasma kallikrein level (median 1.55[IQR 0.87–2.82]) compared to those without the treatment (2.15[1.22–3.84]; p=0.012). When all individuals on RAAS blocker therapy were pooled together (those who used angiotensin-converting-enzyme inhibitors or AT2RBs), then

the plasma kallikrein activity was significantly lower in the individuals receiving therapy (1.72[0.98–3.08]; n=126), compared to those not receiving therapy (2.32[1.26–4.04]; n=157; p=0.016). When data were stratified by DN group, a similar trend remained, but the statistical significance was lost, likely due to the low number of therapy free individuals (data not shown). In unadjusted logistic regression model, plasma kallikrein activity was associated with RAAS-blocker therapy (β=-0.32 [95% CI -0.61 – -0.02], p=0.03).

Finally, we observed, that plasma kallikrein activity, was negatively correlated with systolic blood pressure (r=-0.13, p=0.03) and the association remained after adjustment for age, sex, diabetes duration, waist circumference, HbA1c and RAAS-blocker therapy (β=0.001[95% CI -0.005–0.007], p=0.001).

Plasma kallikrein and genetic variants. The strongest association with plasma kallikrein activity was for *F12* rs1801020 ($p=3.2x10^{-17}$). Homozygote carriers of the rs1801020 minor (A) allele had lower plasma kallikrein activity (AA: median 0.63, IQR [0.40–0.90]; n=16) as opposed to the major (G) allele (GG: 2.72[1.64–4.44]; n=165, Fig. 4a). The rs1801020 SNP accounted for 23% of the variance (r^2=0.23) in the plasma kallikrein activity. The two other SNPs that were associated with kallikrein and FXI activity were rs5030062 and rs710446 near the gene *KNG1* (Table 1). These two SNPs were highly correlated (r^2=0.91) and thus represented the same signal at that locus. Therefore, we focused only on the rs710446 in further analyses (ESM Fig. 2a-b). Altogether, *KNG1* rs710446 and *F12* rs1801020 accounted for 25% of the variance (r^2=0.25) in plasma kallikrein activity. There were no significant SNP-SNP interactions for either plasma kallikrein or FXI activity.

We selected the three SNPs with confirmed associations with plasma kallikrein activity in our primary study cohort of 295 individuals and tested their association with DN, systolic and diastolic blood pressure. Additionally, we tested the SNP association with 24h urine sodium

concentration (N=2675). The Bonferroni corrected statistical significance threshold for the three SNPs, was $p<0.016$.

None of the tested SNPs were associated with systolic blood pressure or DN in linear and logistic regression models, respectively, adjusted for age, sex, diabetes duration, RAAS-blocker therapy and for the principal components. The minor allele of *F12* rs1801020 was associated with 24h urine sodium concentration ($\beta=2.15$, $p=0.03$).

References

1. Levey AS, Stevens LA (2010) Estimating GFR using the CKD Epidemiology Collaboration (CKD-EPI) creatinine equation: more accurate GFR estimates, lower CKD prevalence estimates, and better risk predictions. Am J Kidney Dis Off J Natl Kidney Found 55(4):622–627. https://doi.org/10.1053/j.ajkd.2010.02.337

2. Syreeni A, Sandholm N, Cao J, et al (2019) Genetic Determinants of Glycated Hemoglobin in Type 1 Diabetes. Diabetes db180573. https://doi.org/10.2337/db18-0573

3. Team RC (2013) A language and environment for statistical computing. Vienna, Austria: R Foundation for Statistical Computing. URL Httpwww R-Proj Org

4. Gauderman WJ, Morrison JM, Morrison WGJ (2006) QUANTO 1.1: A computer program for power and sample size calculations for genetic-epidemiology studies

5. Schmaier AH (2003) The kallikrein-kinin and the renin-angiotensin systems have a multilayered interaction. Am J Physiol-Regul Integr Comp Physiol 285(1):R1–R13. https://doi.org/10.1152/ajpregu.00535.2002

6. Schmaier AH (2002) The plasma kallikrein-kinin system counterbalances the renin-angiotensin system. J Clin Invest 109(8):1007–1009. https://doi.org/10.1172/JCI15490

7. Schmaier AH (2014) Physiologic activities of the Contact Activation System. Thromb Res 133:S41–S44. https://doi.org/10.1016/j.thromres.2014.03.018

8. Su JB (2014) Different cross-talk sites between the renin–angiotensin and the kallikrein–kinin systems. J Renin Angiotensin Aldosterone Syst 15(4):319–328. https://doi.org/10.1177/1470320312474854

9. Biswas N, Maihofer AX, Mir SA, et al (2016) Polymorphisms at the F12 and KLKB1 loci have significant trait association with activation of the renin-angiotensin system. BMC Med Genet 17. https://doi.org/10.1186/s12881-016-0283-5

10. Suhre K, Arnold M, Bhagwat AM, et al (2017) Connecting genetic risk to disease end points through the human blood plasma proteome. Nat Commun 8:14357. https://doi.org/10.1038/ncomms14357

11. Sennblad B, Basu S, Mazur J, et al (2017) Genome-wide association study with additional genetic and post-transcriptional analyses reveals novel regulators of plasma factor XI levels. Hum Mol Genet 26(3):637–649. https://doi.org/10.1093/hmg/ddw401

12. Kanaji T, Okamura T, Osaki K, et al (1998) A Common Genetic Polymorphism (46 C to T Substitution) in the 5′-Untranslated Region of the Coagulation Factor XII Gene Is Associated With Low Translation Efficiency and Decrease in Plasma Factor XII Level. Blood 91(6):2010–2014

Tables

ESM Table 1 *Selected SNPs and their published associations with FXI, FXII and plasma kallikrein protein level*

Chr	SNP	GENE	Target trait	MA	MAF	Effect on trait
3	rs5030062	*KNG1*	Factor XI /Pre-kallikrein	C	0.39	unit increase (FXI), unit increase (Pre-kallikrein)[10]
3	rs710446	*KNG1*	Factor XI	C	0.41	unit increase[11]
4	rs1511802	*KLBK1*	Pre-kallikrein	C	0.37	unit increase[10]
4	rs6842047	*KLKB1*	Factor XI	A	0.11	unit decrease[10]
4	rs4253417	*F11*	Factor XI	C	0.41	unit increase [11]
4	rs2289252	*F11*	Factor XI	T	0.41	unit increase[10]
5	rs1801020	*F12*	Factor XII	A	0.20	unit decrease[12]

Chr=Chromosome, MA=Minor allele, MAF=Minor allele frequency

ESM Table 2 *Genetic characteristics of the selected SNPs*

Chr	SNP	Gene	MA	OA	MAF_{subset}	P_{HWE_subset}	MAF_{all}	P_{HWE_all}
3	rs5030062	*KNG1*	C	A	0.35	0.80	0.34	0.43
3	rs710446	*KNG1*	C	T	0.37	0.71	0.37	0.77
4	rs1511802	*KLKB1*	C	T	0.44	0.48	0.43	0.95
4	rs6842047	*KLKB1*	A	C	0.11	0.23	0.09	0.4
4	rs4253417	*F11*	C	T	0.45	0.34	0.44	0.47
4	rs2289252	*F11*	T	C	0.44	0.34	0.43	0.58
5	rs1801020	*F12*	A	G	0.25	0.64	0.26	0.54

Chr=Chromosome, MA=Minor allele, MAF=Minor allele frequency, OA=other allele, HWE=Hardy-Weinberg equilibrium. Number of patients in subset 294, number of all patients=4400

ESM Table 3 *Patient characteristics of the plasma measurement cohort*

	Individuals with type 1 diabetes				
	normo-albuminuria	micro-albuminuria	macro-albuminuria	ESRD	p-value
**Subjects, n (M/F)**	165 (79/86)	41 (20/21)	37 (26/11)	52 (38/14)	
**Age (years)**	46.0±13.5	49.8±12.4	51.2±10.1	45.9±9.7	0.051
**Diabetes duration (years)**	27.3±12.2	35.0±11.3	36.2±10.3	34.5±8.4	<0.001
**Systolic blood pressure (mmHg)**	134±17	138±17	147±23	141±21	<0.001
**Diastolic blood pressure (mmHg)**	75±9	73±8	77±12	77±12	0.289
**Medication, yes (%)**					
**ACE-inhibitors***	21 (13.0)	12 (29.3)	13 (36.1)	12 (23.5)	<0.001
**AT2RBs***	24 (14.8)	22 (53.7)	22 (61.1)	12 (23.5)	<0.001
**Warfarin**	2 (1.2)	2 (4.9)	1 (2.8)	1 (2.0)	0.001
**Laboratory parameters**					
**eGFR (mL/min/1.73m^2) (median [IQR])**	105 [91, 112]	90 [71, 106]	48 [28, 78]	46 [11, 75]	<0.001
**AER (mg/24h)**	6.7 [3.9, 10.2]	42.2 [10.3, 152]	637 [243, 1812]	-	<0.001
data available, n	90	29	23	-	
**HbA_{1c} (mmol/mol) (mean ± sd)**	64±12	69±11	67±13	60±18	0.004
**HbA_{1c} *(%)* (mean ± sd)**	8±3.2	8.5±3.2	8.3±3.3	7.6±3.8	0.004
**Coagulation factors**					
**Plasma kallikrein activity (RU) (median [IQR])**	2.60 [1.40, 4.10]	2.04 [1.22, 3.35]	1.44 [0.96, 2.83]	1.35 [0.72, 1.72]	<0.001
**Plasma factor XI activity (U/mL) (mean ± sd)**	0.97±0.18	0.96±0.15	0.99±0.13	0.88±0.16	0.004
**Genotype distribution**					
F12 rs1801020, n(%)					
AA	8 (4.8)	0 (0)	5 (13.5)	3 (5.7)	
AG	60 (36.4)	14 (34.2)	17 (46)	23 (44.3)	
GG	97 (58.8)	27 (65.8)	15 (40.5)	25 (50)	
KNG1 rs710446, n(%)					
TT	67 (40.6)	16 (39)	13 (35.2)	22 (42.3)	
CT	77 (46.7)	18 (43.9)	16 (43.2)	23 (44.2)	
CC	21 (12.7)	7 (17.1)	8 (21.6)	7 (13.5)	

** ACE – Angiotensin-converting enzyme; AT2RB – Angiotensin-2 receptor blocker; ESRD – end-stage renal disease, IQR – interquartile range; RU – relative unit, AER – albumin excretion rate, ACR – albumin creatinine ratio*

Figures

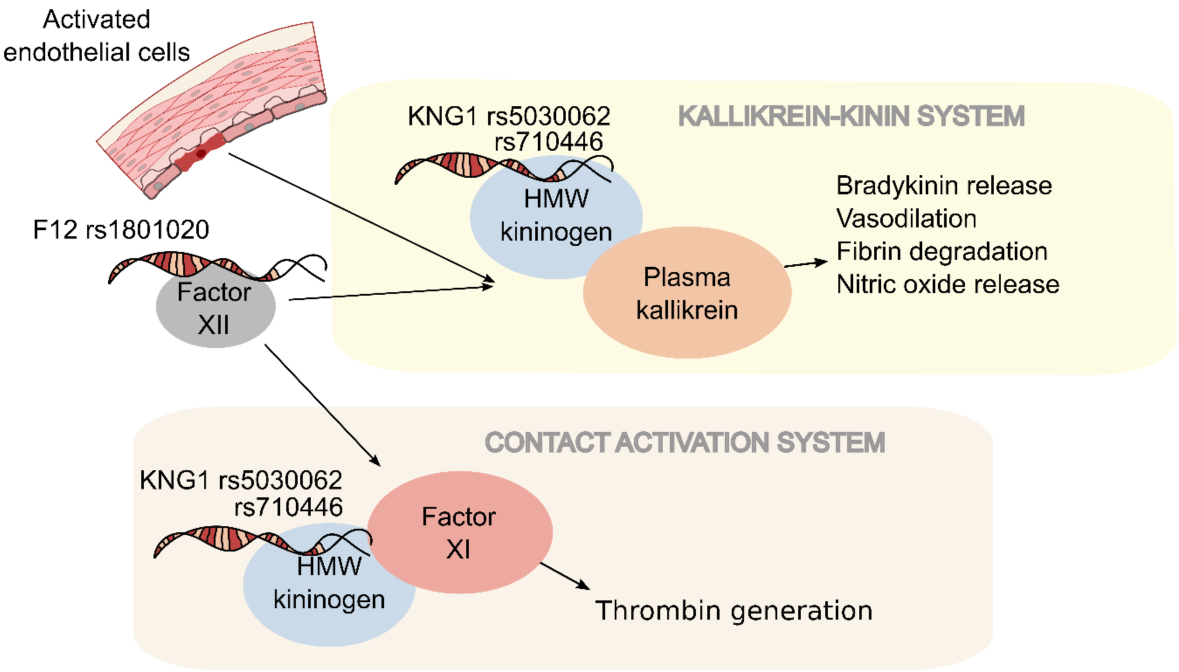

ESM Fig. 1 *The activation of the kallikrein-kinin system. The kallikrein-kinin system is a group of plasma proteins that are activated by FXII or by contact with the activated endothelial surface. Plasma kallikrein activation results in the liberation of bradykinin from high molecular weight (HMW) kininogen, which in turn acts as a vasodilator. Also, activated plasma kallikrein participates in fibrin degradation and nitric oxide release. FXII-mediated cleavage of Factor XI leads to thrombin activation and clotting.*

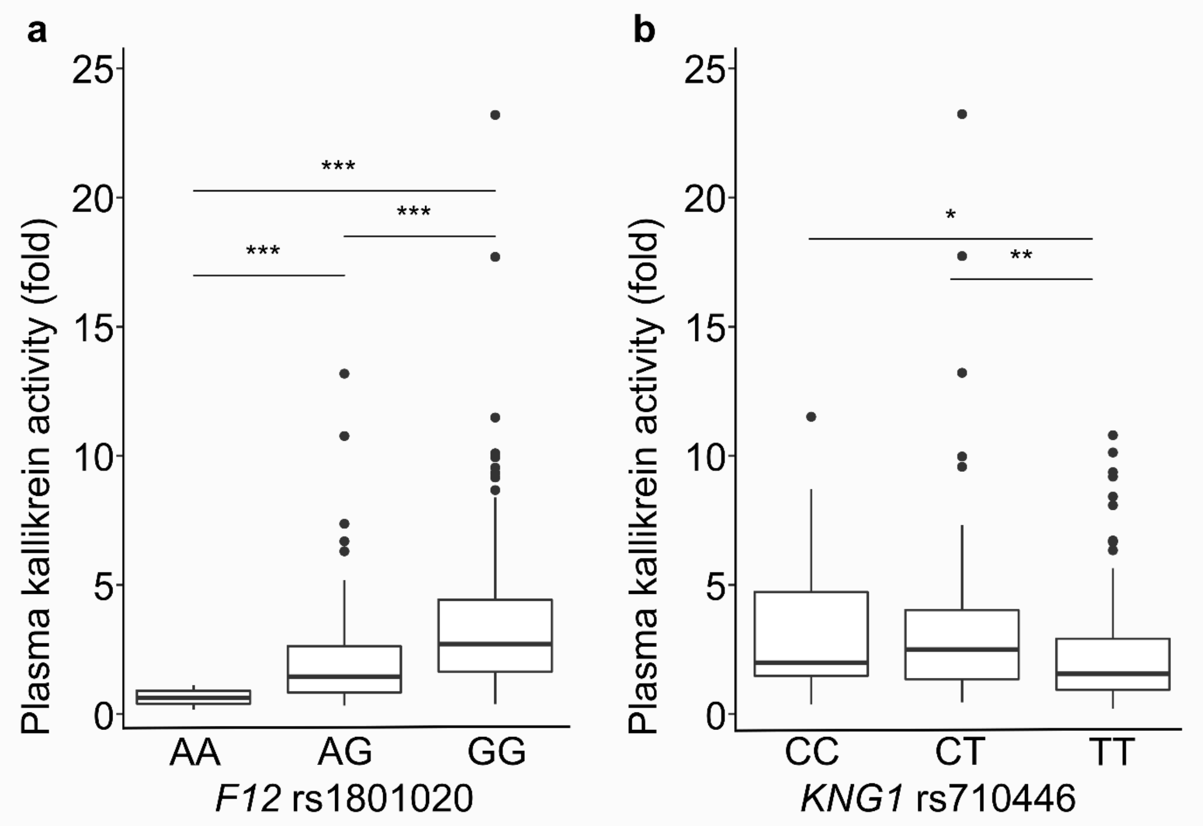

ESM Fig. 2 (a) *Plasma kallikrein activity differs by genotypes of common genetic variants in (2a) factor XII (F12, rs1801020) and* **(b)** *high-molecular-weight kininogen (KNG1, rs710446) genes. In addition to KNG1 rs710446, also KNG1 rs5030062 was associated with plasma kallikrein activity. Because these two SNPs were highly correlated (r2=0.91) and thus represented the same signal at that locus, only rs710446 is represented in this figure. F12, rs1801020: AA, n=16; AG, n=114; GG, n=164. KNG1, rs710446: CC, n=43; CT, n=134; TT, n=118. Plasma kallikrein activity is expressed as fold change against healthy control plasma samples. Boxplots represent median, inter-quartile range and upper and lower quartile. *p< 0.05, **p<0.01, ***p<0.001*

Appendix. *Physicians and nurses at health care centres participating in the collection of FinnDiane patients.*

FinnDiane Study Centers	Physicians and nurses
Anjalankoski Health Centre	S. Koivula, T. Uggeldahl
Central Finland Central Hospital, Jyväskylä	T. Forslund, A. Halonen, A. Koistinen, P. Koskiaho, M. Laukkanen, J. Saltevo, M. Tiihonen
Central Hospital of Åland Islands, Mariehamn	M. Forsen, H. Granlund, A-C. Jonsson, B. Nyroos
Central Hospital of Kanta-Häme, Hämeenlinna	P. Kinnunen, A. Orvola, T. Salonen, A. Vähänen
Central Hospital of Länsi-Pohja, Kemi	H. Laukkanen, P. Nyländen, A. Sademies
Central Ostrabothnian Hospital District, Kokkola	S. Anderson, B. Asplund, U. Byskata, P. Liedes, M. Kuusela, T. Virkkala
City of Espoo Health Centre	
Espoonlahti	A. Nikkola, E. Ritola
Tapiola	M. Niska, H. Saarinen
Samaria	E. Oukko-Ruponen, T. Virtanen
Viherlaakso	A. Lyytinen
City of Helsinki Health Centre	
Puistola	H. Kari, T. Simonen
Suutarila	A. Kaprio, J. Kärkkäinen, B. Rantaeskola
Töölö	P. Kääriäinen, J. Haaga, A-L. Pietiläinen
City of Hyvinkää Health Centre	S. Klemetti, T. Nyandoto, E. Rontu, S. Satuli-Autere
City of Vantaa Health Centre	
Korso	R. Toivonen, H. Virtanen
Länsimäki	R. Ahonen, M. Ivaska-Suomela, A. Jauhiainen
Martinlaakso	M. Laine, T. Pellonpää, R. Puranen
Myyrmäki	A. Airas, J. Laakso, K. Rautavaara
Rekola	M. Erola, E. Jatkola
Tikkurila	R. Lönnblad, A. Malm, J. Mäkelä, E. Rautamo
Heinola Health Centre	P. Hentunen, J. Lagerstam
Helsinki University Central Hospital, Department of Medicine, Division of Nephrology	A. Ahola, J. Fagerudd, M. Feodoroff, D. Gordin, O. Heikkilä, K Hietala, L. Kyllönen, J. Kytö, S. Lindh, K. Pettersson-Fernholm, M. Rosengård-Bärlund, M. Rönnback, A. Sandelin, A-R Salonen, L. Salovaara, L. Thorn, J. Tuomikangas, T. Vesisenaho, J. Wadén
Herttoniemi Hospital, Helsinki	V. Sipilä
Hospital of Lounais-Häme, Forssa	T. Kalliomäki, J. Koskelainen, R. Nikkanen, N. Savolainen, H. Sulonen, E. Valtonen
Iisalmi Hospital	E. Toivanen
Jokilaakso Hospital, Jämsä	A. Parta, I. Pirttiniemi

**FinnDiane Study Centers**	**Physicians and nurses**
Jorvi Hospital, Helsinki University Central Hospital	S. Aranko, S. Ervasti, R. Kauppinen-Mäkelin, A. Kuusisto, T. Leppälä, K. Nikkilä, L. Pekkonen
Jyväskylä Health Centre, Kyllö	K. Nuorva, M. Tiihonen
Kainuu Central Hospital, Kajaani	S. Jokelainen, P. Kemppainen, A-M. Mankinen, M. Sankari
Kerava Health Centre	H. Stuckey, P. Suominen
Kirkkonummi Health Centre	A. Lappalainen, M. Liimatainen, J. Santaholma
Kivelä Hospital, Helsinki	A. Aimolahti, E. Huovinen
Koskela Hospital, Helsinki	V. Ilkka, M. Lehtimäki
Kotka Heath Centre	E. Pälikkö-Kontinen, A. Vanhanen
Kouvola Health Centre	E. Koskinen, T. Siitonen
Kuopio University Hospital	E. Huttunen, R. Ikäheimo, P. Karhapää, P. Kekäläinen, M. Laakso, T. Lakka, E. Lampainen, L. Moilanen, L. Niskanen, U. Tuovinen, I. Vauhkonen, E. Voutilainen
Kuusamo Health Centre	T. Kääriäinen, E. Isopoussu
Kuusankoski Hospital	E. Kilkki, I. Koskinen, L. Riihelä
Laakso Hospital, Helsinki	T. Meriläinen, P. Poukka, R. Savolainen, N. Uhlenius
Lahti City Hospital	A. Mäkelä, M. Tanner
Lapland Central Hospital, Rovaniemi	L. Hyvärinen, S. Severinkangas, T. Tulokas
Lappeenranta Health Centre	P. Linkola, I. Pulli
Lohja Hospital	T. Granlund, M. Saari, T. Salonen
Loimaa Health Centre	A. Mäkelä, P. Eloranta
Länsi-Uusimaa Hospital, Tammisaari	I-M. Jousmaa, J. Rinne
Malmi Hospital, Helsinki	H. Lanki, S. Moilanen, M. Tilly-Kiesi
Mikkeli Central Hospital	A. Gynther, R. Manninen, P. Nironen, M. Salminen, T. Vänttinen
Mänttä Regional Hospital	I. Pirttiniemi, A-M. Hänninen
North Karelian Hospital, Joensuu	U-M. Henttula, P. Kekäläinen, M. Pietarinen, A. Rissanen, M. Voutilainen
Nurmijärvi Health Centre	A. Burgos, K. Urtamo
Oulankangas Hospital, Oulainen	E. Jokelainen, P-L. Jylkkä, E. Kaarlela, J. Vuolaspuro
Oulu Health Centre	L. Hiltunen, R. Häkkinen, S. Keinänen-Kiukaanniemi
Oulu University Hospital	R. Ikäheimo
Päijät-Häme Central Hospital	H. Haapamäki, A. Helanterä, S. Hämäläinen, V. Ilvesmäki, H. Miettinen
Palokka Health Centre	P. Sopanen, L. Welling
Pieksämäki Hospital	V. Javtsenko, M. Tamminen
Pietarsaari Hospital	M-L. Holmbäck, B. Isomaa, L. Sarelin
Pori City Hospital	P. Ahonen, P. Merensalo, K. Sävelä
Porvoo Hospital	M. Kallio, B. Rask, S. Rämö
Raahe Hospital	A. Holma, M. Honkala, A. Tuomivaara, R. Vainionpää
Rauma Hospital	K. Laine, K. Saarinen, T. Salminen

FinnDiane Study Centers	Physicians and nurses
Riihimäki Hospital	P. Aalto, E. Immonen, L. Juurinen
Salo Hospital	A. Alanko, J. Lapinleimu, P. Rautio, M. Virtanen
Satakunta Central Hospital, Pori	M. Asola, M. Juhola, P. Kunelius, M-L. Lahdenmäki, P. Pääkkönen, M. Rautavirta
Savonlinna Central Hospital	E. Korpi-Hyövälti, T. Latvala, E. Leijala
South Karelia Central Hospital, Lappeenranta	T. Ensala, E. Hussi, R. Härkönen, U. Nyholm, J. Toivanen
Tampere Health Centre	A. Vaden, P. Alarotu, E. Kujansuu, H. Kirkkopelto-Jokinen, M. Helin, S. Gummerus, L. Calonius, T. Niskanen, T. Kaitala, T. Vatanen
Tampere University Hospital	I. Ala-Houhala, T. Kuningas, P. Lampinen, M. Määttä, H. Oksala, T. Oksanen, K. Salonen, H. Tauriainen, S. Tulokas
Tiirismaa Health Centre, Hollola	T. Kivelä, L, Petlin, L. Savolainen
Turku Health Centre	I. Hämäläinen, H. Virtamo, M. Vähätalo
Turku University Central Hospital	K. Breitholz, R. Eskola, K. Metsärinne, U. Pietilä, P. Saarinen, R. Tuominen, S. Äyräpää
Vaajakoski Health Centre	K. Mäkinen, P. Sopanen
Valkeakoski Regional Hospital	S. Ojanen, E. Valtonen, H. Ylönen, M. Rautiainen, T. Immonen
Vammala Regional Hospital	I. Isomäki, R. Kroneld, M. Tapiolinna-Mäkelä
Vaasa Central Hospital	S. Bergkulla, U. Hautamäki, V-A. Myllyniemi, I. Rusk
